# Legume allergy: clinical threshold doses in legume-allergic individuals

**DOI:** 10.1186/2045-7022-3-S3-P141

**Published:** 2013-07-25

**Authors:** BC Remington, WM Blom, GF Houben, AC Knulst, JL Baumert, SL Taylor

**Affiliations:** 1 Food Allergy Research & Resource Program, Department of Food Science & Technology, University of Nebraska-Lincoln, Lincoln, NE, USA; 2 Food and Nutrition, TNO, Zeist, Germany; 3 Department of Dermatology/Allergology, University Medical Center Utrecht, Utrecht, the Netherlands

## Background

Soybean and peanut, members of the Legume family, are recognized as common allergenic foods by the FAO Codex Alimentarius. EU directive 2003/89/EC and US Food Allergen Labeling and Consumer Protection Act prescribe labelling of food products for these two major allergenic foods. EU directive 2007/68/EC requires labelling for lupin, another member of the Legume family. The reported prevalence and severity of allergic reactions to soybeans is lower than peanut and most other major allergenic foods. For lupin only limited clinical data are available. Our previous work with peanut has used clinical results from double-blind, placebo-controlled food challenges (DBPCFC) to estimate a population threshold. Further exploration of soy and lupin thresholds would benefit all stakeholders.

## Methods

Publications were screened for DBPCFCs providing lowest observed adverse effect level (LOAEL) and/or no observed adverse effect level (NOAEL) for soy- or lupin-allergic individuals. Unpublished data were retrieved from diagnostic DBPCFCs from the UMCU outpatient population. LOAELs were based upon elicitation of objective symptoms. All doses were converted to mg protein to standardize challenge materials across studies. Individual thresholds were analyzed using Interval-Censoring Survival Analysis and fitted to parametric models (Log-Normal, Log-Logistic, and Weibul) using LIFEREG procedure (SAS v9.2). The eliciting dose predicted to provoke reactions in a proportion of the allergic population(EDp) was estimated and compared to the known peanut-allergic population.

## Results

Public literature revealed clinical thresholds of 43 soy-allergic individuals in 4 studies and 9 lupin-allergic individuals in 3 studies. An additional 15 unpublished lupin DBPCFC were retrieved. Comparison of the ED doses indicate that soy and lupin are less potent allergenic foods than peanut. Using the Log-Normal distributions, the ED_05_ for peanut is 1.3 mg peanut protein while the ED_05_ for lupin is 19.1 mg lupin protein and soybean is 24.5 mg soy protein. However, the lowest eliciting dose observed for each group of allergic individuals is 36 mg lupin protein and 88 mg soy protein, considerably higher than the predicted ED_05_.

## Conclusion

The eliciting dose for allergic reactions of three members of the Legume family showed a different potency for peanut, lupin, and soybean. Additional clinical data from soy and/or lupin challenges would be valuable to increase the statistical confidence in the population threshold estimates.

## Disclosure of interest

None declared.

